# The impact of career adapt-abilities on AI anxiety among English majors: a dual perspective analysis based on core self-evaluations at the person- and variable-centered

**DOI:** 10.3389/fpsyg.2026.1767791

**Published:** 2026-02-20

**Authors:** Xiaoyu Wang

**Affiliations:** School of Foreign Languages, China West Normal University, Nanchong, Sichuan, China

**Keywords:** AI anxiety, career adapt-abilities, core self-evaluations, English majors, variable-centered and person-centered

## Abstract

The rapid advancement of artificial intelligence (AI) technologies in language services, education, and knowledge production has imposed substantial occupational displacement pressures on English majors, thereby triggering significant AI-related anxiety. However, existing research rarely systematically explores the formation mechanisms of AI anxiety among English majors, especially lacking an in-depth analysis of the protective role of career adapt-abilities and their internal heterogeneity. This study adopts a dual-perspective approach—integrating variable-centered and person-centered analyses—to investigate how career adapt-abilities influence AI anxiety and the mediating role of core self-evaluations. A total of 444 English major students from four comprehensive universities in Sichuan, China, were recruited during July and August 2025. Measurements included the Career Adapt-Abilities Scale, Core Self-Evaluation Scale, and AI Anxiety Scale. Results show that career adapt-abilities significantly and negatively predict AI anxiety, with core self-evaluations partially mediating this relationship. Latent profile analysis identified three distinct career adapt-abilities subgroups—low, medium, and high—with significant differences in core self-evaluations and AI anxiety levels among them. Notably, the low career adapt-abilities group exhibited the highest AI anxiety, while the high group showed the lowest. Both analytic strategies converge to demonstrate that career adapt-abilities constitute an essential psychological resource mitigating AI anxiety in English majors, with core self-evaluations serving as a key cognitive mechanism. This study reveals a dual-pathway influence of career adapt-abilities on AI anxiety, offering a novel theoretical framework for understanding technological anxiety formation. Moreover, the pronounced heterogeneity of career adapt-abilities underscores the necessity for stratified career development education and psychological interventions tailored to diverse student groups, providing practical guidance for optimizing talent cultivation in English major programs.

## 1 Introduction

The rapid evolution of artificial intelligence (AI) technology is reshaping the global labor market landscape, particularly impacting sectors that heavily rely on language skills, such as education, language services, and knowledge work (Frank et al., 2019; da Silva, 2024). Next-generation intelligent tools, represented by large language models like ChatGPT, DeepL, and DeepSeek, as well as neural machine translation systems, have demonstrated text generation, translation, and writing assistance capabilities that approach or even surpass human performance (Son and Kim, 2023; Dabre et al., 2020). This technological breakthrough threatens the long-standing linguistic skill advantage possessed by English major graduates, imposing unprecedented substitution pressures on traditional roles (Crawford et al., 2024; Popenici and Kerr, 2017). The World Economic Forum’s 2025 Future of Jobs Report categorizes positions such as translation, secretarial work, and content editing, commonly linked to English majors, as highly susceptible to automation (Alharbi, 2024). As imminent workforce entrants, English majors are especially sensitive to the occupational uncertainty brought by AI technologies, which may provoke specific psychological stress responses and anxiety, with AI anxiety being the most prominent manifestation (Yaşar and Karagucuk, 2025). AI anxiety has been shown to significantly influence learning engagement, skill development planning, career decision-making, and psychological well-being (Eren et al., 2025; Zhang and Cao, 2025). Despite these implications, existing literature predominantly centers on technology professionals, with insufficient attention to the unique contexts of English majors regarding professional identity, employment pathways, and technological substitution risks. Therefore, systematically investigating the formation mechanisms and influencing factors of AI anxiety within this population holds both theoretical significance and practical implications for optimizing English major talent development models.

### 1.1 Research questions

Given the above problem statement, this study aims to address the following research questions from both variable-centered and person-centered perspectives:

*RQ1*: Do career adapt-abilities significantly and negatively predict AI anxiety among English majors?

*RQ2*: Do core self-evaluations mediate the relationship between career adapt-abilities and AI anxiety?

*RQ3*: Can distinct latent subgroups of career adapt-abilities be identified among English majors?

*RQ4*: Do these subgroups differ significantly in their levels of core self-evaluations and AI anxiety?

By addressing these questions, this study seeks to provide a comprehensive understanding of both the general mechanisms and individual heterogeneity underlying AI anxiety formation among English majors.

### 1.2 Conceptual framework

#### 1.2.1 AI anxiety and its conceptual boundaries

AI anxiety is conceptualized as a domain-specific form of technology-related anxiety, characterized by persistent worry and discomfort triggered by perceived threats posed by AI systems—particularly their substitutive potential, autonomous capability, and rapid performance improvements (Frenkenberg and Hochman, 2025; Li and Huang, 2020). Unlike computer anxiety, which primarily reflects fear or inadequacy in operating computers and related hardware/software (Smit et al., 2025; Igbaria, 1993), AI anxiety is less about device use per se and more about the implications of AI capability for one’s competence and vocational value. AI anxiety also overlaps with but is conceptually narrower than automation anxiety, which broadly concerns job loss due to mechanization and technological change (Kim et al., 2025; Bassett and Roberts, 2023); AI anxiety emphasizes the distinctive features of contemporary AI (e.g., generative capacity, language proficiency, and perceived “human-like” performance) that intensify concerns about replaceability in knowledge and language work (Caporusso, 2023; Jabali et al., 2025).

Consistent with prior conceptualizations, AI anxiety in this study is treated as an occupation-relevant, context-specific psychological response, comprising (a) cognitive concerns about competence obsolescence and replaceability, (b) negative affect (e.g., tension and fear) when encountering AI-related information, and (c) behavioral tendencies such as avoidance or compensatory overreliance on AI tools. This positioning aligns with our measurement approach (AI Anxiety Scale capturing learning-, replacement-, and configuration-related anxieties) and with the focal population of English majors whose anticipated career paths are closely exposed to AI-mediated language services.

#### 1.2.2 AI anxiety: definition and relevance to English majors

The AI anxiety is a specific form of technology anxiety (Johnson and Verdicchio, 2017), referring to persistent worry and discomfort arising from individuals’ perception that AI threatens their occupational value, competence requirements, or sense of meaningful existence (Li and Huang, 2020; Salimi et al., 2025). Compared to general technology anxiety, AI anxiety involves excessive cognitive appraisal of AI capabilities and repeated contemplation of one’s replaceability (Salimi et al., 2025); emotional responses such as tension, fear, and helplessness when confronted with AI-related information (Frenkenberg and Hochman, 2025); and behavioral patterns that oscillate between avoidance of and overreliance on AI tools (Kim et al., 2025). For English majors, AI anxiety exhibits a clear occupational orientation, mainly stemming from anticipatory concerns about automation displacing language service positions (Kelan, 2023). If left unregulated, this anxiety can reduce professional learning investment, disrupt career planning, and impair mental health (Kim and Lee, 2025; Cramarenco et al., 2023). However, most prior studies remain descriptive and lack exploration of protective factors and their mechanisms.

#### 1.2.3 Career adapt-abilities as psychological resources

Career adapt-abilities, understood as key psychological resources enabling individuals to effectively manage career transitions, handle occupational stress, and achieve vocational development (Akturan et al., 2025; Johnston, 2018), are widely regarded as important buffers against technological uncertainty and occupational threats (Zhang et al., 2019; Chen et al., 2020). Career construction theory conceptualizes career adapt-abilities as the readiness and capacity to cope with vocational tasks, transitions, or challenges, encompassing four dimensions: concern, control, curiosity, and confidence (Öztemel and Akyol, 2021; Savickas and Porfeli, 2012). Within the context of AI’s deep integration into language services and education, English majors with high career adapt-abilities are more likely to proactively understand technological trends, adjust occupational positioning, and adopt active skill development strategies, thereby alleviating anxiety driven by anticipated technological substitution (Cheng et al., 2025; Wang et al., 2024). Empirical evidence consistently shows significant negative correlations between career adapt-abilities and adverse psychological outcomes such as career anxiety and burnout (Akturan et al., 2025; Wu et al., 2025), alongside positive associations with career satisfaction and subjective well-being. As a dynamic and cultivable psychological resource, career adapt-abilities are especially critical for English majors during key periods of professional identity formation. Hence, this study seeks to address: (1) whether career adapt-abilities negatively correlate with AI anxiety among English majors, and (2) the internal mechanisms underlying this relationship.

#### 1.2.4 Core self-evaluations as a mediating mechanism

Core self-evaluations refer to fundamental, subconscious assessments individuals make regarding their self-worth, competence, and locus of control (Johnson et al., 2016; Chang et al., 2012). Compared to single personality traits, core self-evaluations represents a higher-order construct with greater explanatory power for career development, mental health, and subjective well-being outcomes (Judge et al., 2003). For English majors, core self-evaluations not only reflects overall judgments of language proficiency and professional competence but also deeply shapes core beliefs about self-worth and irreplaceability amid the AI era (Wang et al., 2021). Drawing on conservation of resources theory, career adapt-abilities as psychosocial resources must undergo specific cognitive processing to exert their protective function (Gupta and Shagirbasha, 2025). Hobfoll (2002) emphasize that resources do not automatically yield adaptive outcomes; their utility depends on individuals’ subjective evaluation and ability to transform resources into coping actions. Core self-evaluations serve as a critical cognitive mediator in this process. Specifically, individuals with high career adapt-abilities accumulate positive experiences and resources, which gradually internalize into stable self-affirmations and self-efficacy, fostering a core belief in their capability to navigate vocational challenges. This positive self-assessment acts as a cognitive shield against external threat information, helping maintain emotional stability when facing AI-induced disruptions. In other words, career adapt-abilities indirectly buffer AI anxiety by shaping and reinforcing core self-evaluations.

#### 1.2.5 The need for dual analytical perspectives

Methodologically, prior research predominantly uses variable-centered approaches, such as regression or structural equation modeling, to explore overall linear relationships among variables (Woo et al., 2024). While effective at revealing general trends, these methods assume sample homogeneity and may overlook internal heterogeneity. Individuals are not mere linear sums of variables; career adapt-abilities and core self-evaluations may manifest in diverse, complex patterns across different individuals. For example, some students might show atypical profiles like “high career concern but low confidence,” whose unique psychological mechanisms could be masked by average effects. Sole reliance on variable-centered perspectives risks ecological validity and limits precise guidance for differentiated educational interventions. To address this gap, this study innovatively incorporates a person-centered approach via latent profile analysis to identify distinct career adapt-abilities types among English majors, complemented by variable-centered analyses to construct a comprehensive, dual-perspective analytical framework for richer theoretical insights.

### 1.3 Research hypotheses

Based on the conceptual framework outlined above, the present study proposes the following hypotheses:

*H1*: Career adapt-abilities significantly and negatively predict AI anxiety among English majors.

*H2*: Core self-evaluations partially mediate the relationship between career adapt-abilities and AI anxiety.

*H3*: Distinct latent subgroups of career adapt-abilities exist among English majors, reflecting qualitatively different configurations of career psychological resources.

*H4*: These latent subgroups exhibit significant differences in core self-evaluations and AI anxiety levels.

### 1.4 Significance of the study

Based on this theoretical foundation, the present study proposes an integrative model. From the variable-centered viewpoint, career adapt-abilities are hypothesized to significantly and negatively predict AI anxiety, with core self-evaluations mediating this effect. From the person-centered perspective, we hypothesize the existence of qualitatively distinct career adapt-abilities profiles exhibiting significant differences in core self-evaluations and AI anxiety levels. Specifically, students with positive career adapt-abilities profiles are expected to possess stronger core self-evaluations and correspondingly lower AI anxiety, whereas those with negative profiles may lack internal psychological support, rendering core self-evaluations less effective in buffering anxiety. This dual-perspective approach not only validates general variable relationships but also reveals individual-specific coping mechanisms, greatly enhancing explanatory power and theoretical depth.

Theoretical contribution. Although Career Construction Theory and Conservation of Resources theory have both been used to explain career-related adjustment and stress responses (Nalis et al., 2022), prior work in technology-related anxiety has seldom articulated how career adapt-abilities become psychologically effective under AI-induced threat. The present study advances an integrative account by specifying a resource–internalization–anxiety pathway: career adapt-abilities (as Career Construction Theory-defined psychosocial resources for managing vocational tasks and transitions) reduce AI anxiety partly through core self-evaluations, which represents the cognitive internalization and appraisal mechanism emphasized in Conservation of Resources theory. In this integrated framework, adapt-abilities are not merely “resources” in a general sense; they are theorized to generate mastery experiences and proactive coping, which are then consolidated into more positive core self-evaluations (e.g., perceived competence, control, and emotional stability). Higher core self-evaluations subsequently lowers AI anxiety by weakening threat appraisal and enhancing perceived controllability when students encounter AI-related occupational uncertainty.

Importantly, this integration yields a testable implication beyond parallel application: the protective effect of career adapt-abilities should be partially indirect (via core self-evaluations) and partially direct, indicating that resources operate through both cognitive internalization (Conservation of Resources Theory) and more immediate adaptive regulation/behavioral readiness (Career Construction Theory). This proposition is evaluated using a dual-analytic strategy (variable-centered mediation and person-centered profiling), thereby linking mechanism-level explanations with heterogeneity in resource configurations.

Practically, the findings provide precise intervention guidance for university administrators and career counselors. By identifying distinct career adapt-abilities profiles among English majors, educators can implement stratified and targeted psychological counseling strategies: for students in the negative profile group, the focus should be on cultivating their career adapt-abilities and reconstructing self-cognition; for those in the positive profile group, efforts should concentrate on consolidating psychological strengths and enhancing their specific human–machine collaboration skills.

### 1.5 Nature of contributions

The primary contribution of this study is theoretical, by articulating and testing an integrated mechanism through which career adapt-abilities buffer AI anxiety via the cognitive resource of core self-evaluations (resource internalization). Second, the study makes a methodological contribution by triangulating findings through variable-centered mediation analysis and person-centered latent profile analysis, enabling simultaneous examination of general mechanisms and within-population heterogeneity. Third, the study offers a contextual contribution by extending AI anxiety research to English majors, a group facing salient AI-driven substitution pressures in language-related occupations, thereby enriching the boundary conditions of existing technology-anxiety frameworks.

## 2 Theoretical background and conceptual constructs

### 2.1 Conservation of resources theory and Hobfoll’s resource model

Conservation of Resources Theory conceptualizes stress as a reaction to (a) the threat of resource loss, (b) actual resource loss, or (c) insufficient resource gain following resource investment (Hobfoll, 1989; Hobfoll and Freedy, 2017). Resources are broadly defined as objects, personal characteristics, conditions, and energies that people value or that serve as means to obtain valued outcomes (Brown, 1984). A central proposition of Hobfoll’s resource model is that individuals strive to acquire, retain, and protect resources; when resources are threatened or depleted, stress and anxiety increase (Hobfoll, 1989). Conservation of Resources Theory also highlights resource caravans (clusters of resources that co-occur) and gain/loss spirals, whereby initial resource deficits make individuals more vulnerable to subsequent losses and negative affect, while resource abundance facilitates further gains (Chen et al., 2015).

In the present study, AI-driven changes in language services constitute an external stressor that may be appraised as a threat to English majors’ valued resources (e.g., employability, occupational identity, perceived competence) (Javaid et al., 2024). Within Conservation of Resources Theory, career adapt-abilities are conceptualized as a set of psychosocial resources that can buffer stress by enabling proactive coping and effective adaptation to occupational transitions (Johnston, 2018). However, Conservation of Resources Theory also suggests that resources exert protective effects through appraisal and internalization processes (Chen et al., 2015); thus, we position core self-evaluations as a key cognitive resource that helps translate career adapt-abilities into lower AI anxiety.

### 2.2 Career construction theory and career adapt-abilities

Career Construction Theory posits that individuals actively construct career paths by adapting to vocational tasks, transitions, and traumas through self-regulatory psychosocial resources (Savickas, 2013). Within this framework, career adapt-abilities represent adaptive capacity and readiness and are commonly operationalized via four dimensions: concern (future orientation and planning), control (self-discipline and decision agency), curiosity (exploration of possible selves and opportunities), and confidence (self-efficacy in solving career-related problems) (Potomkin, 2024). In an AI-disruption context, English majors with higher adapt-abilities are expected to monitor technological trends, reframe occupational threats into development tasks, and engage in upskilling and human–AI collaboration learning. These tendencies can reduce threat appraisal and uncertainty, thereby decreasing AI anxiety. Conversely, low adapt-abilities may reflect limited future planning and coping efficacy, increasing vulnerability to AI-related occupational stressors.

### 2.3 Core self-evaluations as a higher-order self-belief construct

Core self-evaluations describe individuals’ fundamental appraisals of their own worth and competence (Chang et al., 2012). Core self-evaluations is a higher-order trait-like construct typically comprising four elements: self-esteem, general self-efficacy, (low) neuroticism/emotional stability, and locus of control (Chen, 2012). Compared with single traits, core self-evaluations provides a more integrative account of how people interpret challenges, evaluate their coping ability, and regulate affect. In this study, core self-evaluations is theorized as a cognitive mechanism and resource that shapes how English majors interpret AI-related information and whether they perceive themselves as capable of adapting. Under Conservation of Resources Theory, higher career adapt-abilities may facilitate mastery experiences and proactive coping, which can be internalized into more positive core self-evaluations. In turn, higher core self-evaluations should reduce AI anxiety by promoting perceived control and competence under uncertainty.

### 2.4 Locus of control: conceptual role in the present study

Locus of control refers to the extent to which individuals believe outcomes are contingent on their own behavior (internal locus) versus determined by external forces such as luck, fate, or powerful others (external locus) (Galvin et al., 2018). An internal locus is generally associated with stronger perceived agency, more problem-focused coping, and lower stress reactivity in uncertain contexts (Hahn, 2000). In the current manuscript, locus of control is not modeled as an independent variable; instead, it functions as a core component within core self-evaluations (Judge et al., 2003). Conceptually, the “control” component at the core self-evaluations level complements the behavioral-regulatory “control” dimension in career adapt-abilities, together forming a psychological basis for perceiving AI-related disruptions as manageable rather than overwhelming.

## 3 Methods

### 3.1 Research design

This study employs a cross-sectional survey design to systematically examine the relationships among career adapt-abilities, core self-evaluations, and AI anxiety in English majors. It combines variable-centered and person-centered analytical perspectives to achieve broader and deeper theoretical insights. The cross-sectional design enables efficient collection of large-scale data at a single time point, suitable for identifying correlations among variables and constructing mediation models. Within the variable-centered framework, structural equation modeling (SEM) is used to test the mediating pathway “career adapt-abilities → core self-evaluations → AI anxiety,” revealing general causal patterns. Within the person-centered framework, latent profile analysis (LPA) identifies heterogeneous subgroups of career adapt-abilities and compares differences in core self-evaluations and AI anxiety among these profiles. Data were collected via online questionnaires using the professional survey platform “Credamo”^1^, ensuring standardized data collection procedures and quality control.

### 3.2 Ethical considerations

This study strictly adheres to the ethical principles outlined in the Declaration of Helsinki and obtained approval from the Academic Ethics Committee of China West Normal University (Approval No.:2025LLSC0090). During informed consent, all potential participants were required to read detailed study information—including objectives, content, estimated time, potential risks and benefits, data confidentiality, and voluntary participation and withdrawal rights—before proceeding to the formal questionnaire. Participants confirmed their informed consent by electronically ticking an agreement box; those who did not consent could not continue. Participant privacy was strictly protected, with no personal identifying information such as names, student IDs, or contact details collected.

### 3.3 Participants

#### 3.3.1 Recruitment procedure

A stratified convenience sampling strategy was employed to recruit English major undergraduates from four comprehensive universities in Sichuan Province, China, during July and August 2025. To enhance representativeness and heterogeneity, stratification was based on institutional type and academic year. Institutional levels included “Double First-Class” universities, provincial key universities, and ordinary undergraduate institutions. Academic years ranged from freshman to senior undergraduate students.

Recruitment combined online and offline methods. Online channels leveraged university English major student WeChat groups, QQ groups, and professional course platforms to disseminate recruitment information. Offline channels involved English department advisors and instructors promoting the study during classes. Recruitment materials clearly stated the study topic, eligibility criteria, questionnaire duration, and participation incentives. Participants accessed the questionnaire via QR codes or hyperlinks. To improve response rates and data quality, participants who completed the questionnaire received a small gift valued at approximately 3 Chinese yuan as compensation.

#### 3.3.2 Minimum sample size

Sample size requirements were determined through *a priori* power analyses for both analytical methods, with the larger requirement adopted as the minimum standard. For SEM, G*Power software was used with parameters set to a medium effect size (*f*^2^ = 0.15), statistical power (1–*β*) = 0.95, significance level (*α*) = 0.05, and 11 predictors in the regression model, yielding a minimum required sample size of 119. Regarding latent profile analysis, Monte Carlo simulation studies by Nylund et al. (2007) indicate that a sample size exceeding 400 is needed to reliably identify the correct number of profiles through information criteria and likelihood ratio tests. Therefore, this study set a minimum target sample size of 400.

#### 3.3.3 Inclusion and exclusion criteria

To ensure sample appropriateness and measurement accuracy, inclusion criteria were: (1) full-time undergraduate or graduate students majoring in English (including English language and literature, translation, business English, etc.) at regular higher education institutions in mainland China; (2) aged 18 years or older; (3) able to understand the questionnaire content and complete it independently, with sufficient Chinese reading ability; (4) voluntarily participated and signed electronic informed consent.

Exclusion criteria included: (1) non-English majors or students with only minor English study to avoid confounding due to differing professional identity; (2) students currently on leave, withdrawn, or delayed graduation, as their academic status might affect career adapt-abilities and anxiety assessment; (3) students undergoing psychological treatment or using psychiatric medication to minimize clinical interference in anxiety and self-evaluation measurements; (4) respondents exhibiting obvious response pattern regularities indicating inattentive answering; (5) questionnaires completed in excessively short or long times indicating careless or distracted responses.

#### 3.3.4 Final sample size

A total of 500 questionnaires were distributed, with 489 returned, representing a 97.8% response rate. During data cleaning, 16 respondents under 18 years old, 19 minor English majors, and 10 questionnaires with highly regular response patterns were excluded. Ultimately, 45 invalid questionnaires were removed, resulting in a valid sample size of 444 and an effective rate of 90.79%.

### 3.4 Measurement instruments

#### 3.4.1 Career adapt-abilities scale

The Career Adapt-Abilities Scale used in this study originates from the Chinese version developed by Hou et al. (2012). It consists of 24 items across four dimensions: concern (6 items), control (6 items), curiosity (6 items), and confidence (6 items). Originally validated with Chinese university students, the scale demonstrated cultural adaptability and reliability. This study measured career adapt-abilities of English majors using a 5-point Likert scale ranging from 1 (strongly disagree) to 5 (strongly agree). For example, one item reads: “Do you think about what your future will look like?” The total score ranges from 24 to 120, with higher scores indicating stronger career adapt-abilities. The validity and reliability of this scale are shown in Table 1.

**Table 1:** Model goodness-of-fit for the core study variables.

Variables	*χ*^2^/df	GFI	AGFI	RMSEA	CFI	TLI	Cronbach’s *α*
Career adapt-abilities	3.406	0.847	0.815	0.074	0.888	0.875	0.944
Core self-evaluation	1.361	0.942	0.896	0.029	0.984	0.980	0.879
AI anxiety	1.520	0.967	0.957	0.034	0.988	0.987	0.936

#### 3.4.2 Core self-evaluation scale

The Core Self-Evaluation Scale was developed by Judge et al. (2003) and translated into Chinese by Sun and Jiang (2017), with demonstrated cultural validity and reliability in Chinese adult populations. It contains 12 items representing a single dimension, reflecting four traits: self-esteem, general self-efficacy, neuroticism, and locus of control. The scale uses a 5-point Likert rating from 1 (strongly disagree) to 5 (strongly agree). An example item is: “Do you agree that you are confident you can achieve the success you deserve?” Scores range from 12 to 60, with higher scores indicating higher core self-evaluation ability. The validity and reliability of this scale are shown in Table 1.

#### 3.4.3 AI anxiety scale

The AI Anxiety Scale, developed by Wang and Wang (2022), consists of 21 items divided into four dimensions: learning (8 items), job replacement (6 items), social-technical blind spots (4 items), and AI configuration (3 items). Widely applied among Chinese student groups, including Chinese student interpreters (Zhong and Ma, 2025), this scale uses a 5-point Likert scale from 1 (strongly disagree) to 5 (strongly agree). For example: “Do you agree that learning all the special features of AI technology/products makes you anxious?” Scores range from 21 to 105, with higher scores indicating greater AI anxiety. The validity and reliability of this scale are shown in Table 1.

### 3.5 Statistical methods

Data analysis proceeded through three phases using SPSS 27.0, Mplus 8.3, Amos 30.0 and SPSS PROCESS macro version 4.1.

Phase 1: Preliminary Analysis. Descriptive statistics, including means, standard deviations, skewness, and kurtosis, were computed for all variables. Pearson correlations were calculated to examine bivariate relationships. Confirmatory factor analysis (CFA) was conducted to verify the measurement model among the three latent constructs.

Phase 2: Variable-Centered Analysis. To test Hypotheses 1 and 2, mediation analysis was performed using the PROCESS macro (Model 4) with 5,000 bootstrap samples to estimate indirect effects and 95% bias-corrected confidence intervals. Career adapt-abilities served as the independent variable, AI anxiety as the dependent variable, and core self-evaluations as the mediator. Gender and academic year were included as covariates.

Phase 3: Person-Centered Analysis. To test Hypotheses 3 and 4, latent profile analysis (LPA) was conducted using Mplus 8.3. The four dimensions of career adapt-abilities (concern, control, curiosity, confidence) served as profile indicators. Models with one through six profiles were estimated and compared using multiple fit indices: Akaike Information Criterion (AIC), Bayesian Information Criterion (BIC), sample-size adjusted BIC (aBIC), entropy, Lo–Mendell–Rubin adjusted likelihood ratio test (LMR-LRT), and bootstrap likelihood ratio test (BLRT). Lower AIC, BIC, and aBIC values indicate better fit; entropy values closer to 1.0 indicate clearer class separation; significant LMR-LRT and BLRT values support the k-class solution over the *k*-1 class solution. Following profile identification, one-way ANOVA and *post hoc* comparisons (Bonferroni correction) were used to examine differences in core self-evaluations and AI anxiety across profiles.

## 4 Results

### 4.1 Demographic characteristics

#### 4.1.1 Mini-summary

The sample was predominantly female and largely composed of first-year undergraduates, reflecting the typical gender distribution of English majors.

A total of 444 valid participants were included; detailed demographics are shown in Table 2. Females constituted the vast majority (*N* = 378, 85.10%), with males numbering 66 (14.90%), consistent with the gender distribution of English majors in Chinese universities. The average age was 19.98 years (SD = 6.931). Grade distribution was as follows: freshmen (*N* = 230, 51.80%), sophomores (*N* = 77, 17.30%), seniors (*N* = 72, 16.20%), juniors (*N* = 45, 10.10%), and graduate students or above (*N* = 20, 4.50%). Rural-origin students (*N* = 283, 63.70%) outnumbered urban-origin students (*N* = 161, 36.30%).

**Table 2:** Demographic information of the participants.

Variable	Items	Frequency (N)	Percentage (%)
Gender	Male	66	14.90%
	Female	378	85.10%
Grade	Freshmen	230	51.80%
	Sophomores	77	17.30%
	Juniors	45	10.10%
	Seniors	72	16.20%
	Graduate students or above	20	4.50%
Place of origin	Rural-origin	161	36.30%
	Urban-origin	283	63.70%
AI usage habits	Preliminary, unsystematic learning experience	150	33.80%
	No exposure or use of AI tools	6	1.40%
	Daily tool	256	57.70%
	Systematic training	32	7.20%
Relevance between future careers and AI technologies	No.	296	66.70%
	Yes.	148	33.30%
Age	19.98 ± 6.931		

Regarding AI usage habits, over half (*N* = 256, 57.70%) used AI solely as a daily tool; about one-third (*N* = 150, 33.80%) had preliminary, unsystematic learning experience; only 32 participants (7.20%) underwent systematic training; and 6 individuals (1.40%) reported no exposure or use of AI tools. On perceptions of the relevance between future careers and AI technologies, approximately two-thirds (*N* = 296, 66.70%) believed their future careers were unrelated to AI, while 148 (33.30%) perceived a connection.

### 4.2 Common method bias analysis

Given that the core variables—career adapt-abilities, core self-evaluation, and AI anxiety—were collected via self-report from the same participants at a single time point, common method bias was a potential concern. Harman’s single-factor test was applied by entering all items into an unrotated exploratory factor analysis to assess the variance explained by the first factor. Ten factors with eigenvalues greater than one were extracted, with the first factor accounting for only 27.724% of the variance, below the 40% threshold. Thus, common method bias was not a serious issue in this dataset.

### 4.3 Descriptive statistics and correlation analysis

#### 4.3.1 Mini-summary

Correlations were consistent with H1 and H2: career adapt-abilities were positively associated with core self-evaluations and negatively associated with AI anxiety, supporting further mediation and profile-based analyses.

Table 3 presents descriptive statistics and correlations among the main variables. Normality assumptions were met, with skewness and kurtosis for career adapt-abilities (0.335, 0.660), core self-evaluation (0.558, 1.095), and AI anxiety (−0.54, 0.639) all within acceptable ranges. Mean scores indicated that English majors had moderately high career adapt-abilities (*M* = 3.654, SD = 0.479), reflecting a solid reserve of career coping resources. Core self-evaluation was also moderate to high (*M* = 3.522, SD = 0.405), indicating relatively positive self-worth and efficacy perceptions. AI anxiety averaged 3.275 (SD = 0.686), slightly above the midpoint of the scale, suggesting a moderate level of anxiety about AI among participants.

**Table 3:** Descriptive statistics and correlation analysis of core study variables.

Variables	*M*	SD	Skewness	Kurtosis	1	2	3
1. Career adapt-abilities	3.654	0.479	0.335	0.660			
2. Core self-evaluation	3.522	0.405	0.558	1.095	0.512***		
3. AI anxiety	3.275	0.686	−0.54	0.639	−0.324***	−0.295***	

***p < 0.001.

Correlation analyses aligned with theoretical expectations: career adapt-abilities positively correlated with core self-evaluation (*r* = 0.512, *p* < 0.001), indicating students with stronger career resources tend to have more positive self-evaluations. Career adapt-abilities negatively correlated with AI anxiety (*r* = −0.324, *p* < 0.001), suggesting a protective effect. Core self-evaluation also negatively correlated with AI anxiety (*r* = −0.295, *p* < 0.001). These findings preliminarily support testing the mediation effect.

### 4.4 AI anxiety differences across demographic groups

#### 4.4.1 Mini-summary

Demographic differences in AI anxiety were generally small; grade showed a statistically significant but modest effect. These results justify controlling relevant demographics (e.g., age and grade) in subsequent model tests without suggesting that demographics are primary drivers of AI anxiety in this sample.

Independent samples *t*-tests and one-way ANOVA examined AI anxiety differences across demographic variables (see Table 4). No significant gender differences were found: males (*M* = 3.224, SD = 0.710) and females (*M* = 3.283, SD = 0.682) showed similar AI anxiety levels (*t* = −0.644, *p* = 0.533, Cohen’s *d* = −0.086).

**Table 4:** Tests for differences in AI anxiety on demographic variables (*N* = 444).

Variable	Items	*M*	SD	*t*/*F*	*p*	Cohen *d*/*η*^2^
Gender	Male	3.224	0.710	−0.644	0.533	−0.086
	Female	3.283	0.682			
Grade	Freshmen	3.331	0.646	3.008	0.018	0.027
	Sophomores	3.038	0.763			
	Juniors	3.250	0.797			
	Seniors	3.334	0.630			
	Graduate students or above	3.383	0.599			
Place of origin	Rural-origin	3.250	0.677	−0.571	0.566	−0.056
	Urban-origin	3.289	0.692			
AI usage habits	Preliminary, unsystematic learning experience	3.325	0.693	0.464	0.708	0.003
	No exposure or use of AI tools	3.143	0.656			
	Daily tool	3.253	0.664			
	Systematic training	3.234	0.834			
Relevance between future careers and AI technologies	No.	3.233	0.682	−1.791	0.075	−0.180
	Yes.	3.357	0.688			

Grade-level differences were significant (*F* = 3.008, *p* = 0.018, *η*^2^ = 0.027). *Post hoc* Bonferroni tests revealed sophomores (*M* = 3.038, SD = 0.763) had significantly lower AI anxiety than freshmen (*M* = 3.331, SD = 0.646) and seniors (*M* = 3.334, SD = 0.630). This may reflect developmental differences in career awareness and employment pressure: freshmen face uncertainty entering college, while seniors perceive greater AI-related job threat. However, the effect size was small, indicating limited explanatory power.

No significant differences were found between rural (*M* = 3.289, SD = 0.692) and urban (*M* = 3.250, SD = 0.677) students (*t* = −0.571, *p* = 0.566). AI usage habits also did not significantly affect AI anxiety (*F* = 0.464, *p* = 0.708, *η*^2^ = 0.003), suggesting that familiarity with AI tools alone does not directly influence anxiety levels, which are likely moderated by deeper psychological factors.

Regarding perceptions of future career relevance to AI, those who saw a connection (*M* = 3.357, SD = 0.688) had slightly higher AI anxiety than those who did not (*M* = 3.233, SD = 0.682), but this difference was not statistically significant (*t* = −1.791, *p* = 0.075), showing only a marginal trend.

### 4.5 Variable-centered mediation analysis

#### 4.5.1 Hypothesis reminder (H1–H2)

We tested whether career adapt-abilities negatively predicted AI anxiety (H1) and whether core self-evaluations partially mediated this relationship (H2), controlling for age and grade.

#### 4.5.2 Mini-summary

Results supported H1 and H2. Career adapt-abilities showed a significant negative total and direct effect on AI anxiety, and core self-evaluations contributed a significant partial indirect effect, indicating that career adapt-abilities buffer AI anxiety both directly and via enhanced self-evaluations.

**Figure 1:**
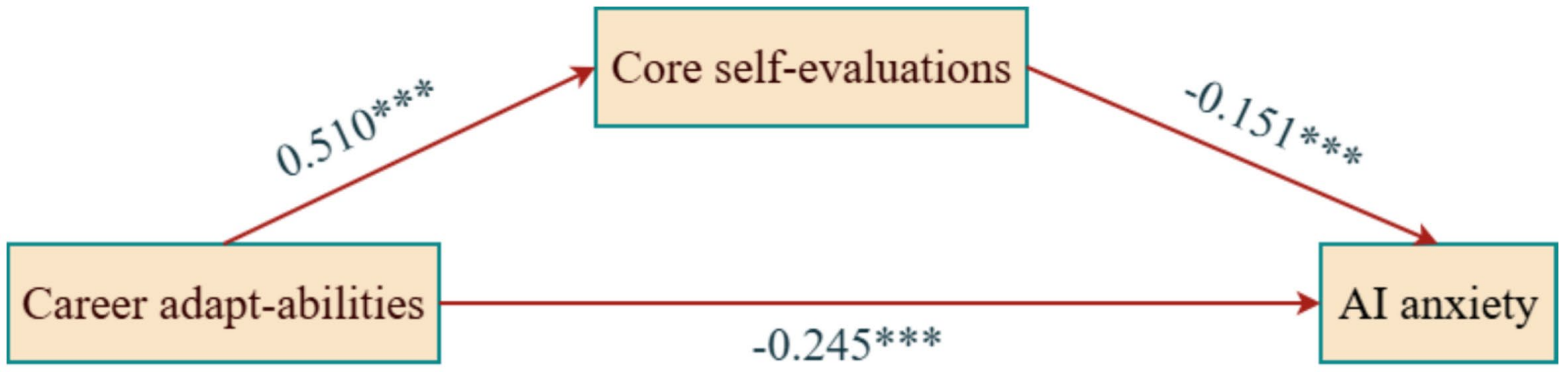
Path coefficients for the mediating effect of core self-evaluation. ***p < 0.001.

Using PROCESS macro (Model 4) in SPSS 27.0, with age and grade controlled, the mediating role of core self-evaluation between career adapt-abilities and AI anxiety was tested via bias-corrected bootstrap (5,000 resamples). Results are detailed in Table 5 and Figure 1.

**Table 5:** Results of regression analysis of mediating effects.

Independent variable	Model 1			Model 2		
	Core self-assessment			AI anxiety		
	*β*	*t*	95%CI	*β*	*t*	95%CI
Career adapt-abilities	0.510	12.514	[0.367, 0.504]	−0.245	−4.709	[−0.499, −0.205]
Core self-assessment				−0.151	−2.891	[−0.429, −0.082]
*R*	0.528			0.376		
*R*^2^	0.279			0.141		
*F*	56.023***			17.827***		

***p < 0.001.

Model 1 regression predicting core self-evaluation showed a significant positive effect of career adapt-abilities (*β* = 0.510, *t* = 12.514, *p* < 0.001, 95% CI [0.367, 0.504]). The model explained 27.9% of variance (*R*^2^ = 0.279, *F* = 56.023, *p* < 0.001), supporting the idea that career adapt-abilities foster positive self-cognition. Model 2 regression predicting AI anxiety included both predictors. Career adapt-abilities directly negatively predicted AI anxiety (*β* = −0.245, *t* = −4.709, *p* < 0.001, 95% CI [−0.499, −0.205]), and core self-evaluation also negatively predicted AI anxiety (*β* = −0.151, *t* = −2.891, *p* < 0.01, 95% CI [−0.429, −0.082]). Together, they accounted for 14.1% of AI anxiety variance (*R*^2^ = 0.141, *F* = 17.827, *p* < 0.001).

Table 6 shows mediation decomposition: total effect of career adapt-abilities on AI anxiety was −0.463 (SE = 0.065, 95% CI [−0.590, −0.336]), significant as CI excludes zero. After including mediator, direct effect remained significant (−0.352, SE = 0.075, 95% CI [−0.499, −0.205]), accounting for 76% of total effect. Indirect effect via core self-evaluation was −0.111 (SE = 0.051, 95% CI [−0.208, −0.007]), showing a significant partial mediation effect (24%). These results indicate core self-evaluation partially mediates the relationship between career adapt-abilities and AI anxiety. Specifically, career adapt-abilities reduce AI anxiety both directly and indirectly by enhancing positive self-evaluation. This aligns with Conservation of Resources Theory: career adapt-abilities as vital psychosocial resources exert protective effects partly through deep self-cognitive structures, helping students internalize positive experiences and form a cognitive shield against AI threat perceptions, resulting in lower anxiety.

**Table 6:** Effect decomposition of the mediating effects of core self-evaluations.

Decomposition of effects	*β*	SE	LLCI	ULCI	Proportion of effect
Total effect	−0.463	0.065	−0.59	−0.336	100%
Direct effect	−0.352	0.075	−0.499	−0.205	76%
Indirect effect	−0.111	0.051	−0.208	−0.007	24%

LLCI = lower limit of 95% confidence interval; ULCI = upper limit of 95% confidence interval; the Bootstrap sample was repeated 5,000 times.

### 4.6 Person-centered analysis

#### 4.6.1 Hypothesis reminder (H3)

To examine heterogeneity beyond average effects, we tested whether distinct latent profiles of career adapt-abilities could be identified among English majors (H3).

#### 4.6.2 Mini-summary

A three-profile solution (low, moderate, and high career adapt-abilities) provided the most parsimonious and interpretable fit, supporting the heterogeneity premise of H3 and motivating comparisons of profile differences in core self-evaluations and AI anxiety.

To identify heterogeneous subgroups of career adapt-abilities among English major students, latent profile analysis (LPA) was conducted using Mplus 8.3. The 24 items of the Career Adapt-Abilities Scale served as profile indicators. Models with 2 to 5 profiles were fitted sequentially; model fit indices are detailed in Table 7.

**Table 7:** Model fit indices for 1- to 5-profile latent profile models of career adapt-abilities.

Profile	AIC	BIC	aBIC	Entropy	LMR (p)	BLRT (p)	Proportion of potential subgroups
1	23373.383	23569.982	23417.651	–	–	–	–
2	20560.075	20859.070	20627.400	0.949	<0.001	<0.001	47.2%/52.8%
3	19431.435	19832.826	19521.817	0.952	0.006	<0.001	41.0%/51.2%/7.8%
4	19077.146	19580.932	19190.584	0.928	0.182	<0.001	15.9%/31.4%/45.4%/7.3%
5	18921.803	19527.985	19058.299	0.923	0.789	<0.001	2.0%/21.2%/26.8%/42.8%/7.1%

AIC = Akaike Information Criterion; BIC = Bayesian Information Criterion; aBIC = Sample-Size Adjusted BIC; LMR = Lo–Mendell–Rubin Adjusted Likelihood Ratio Test; BLRT = Bootstrap Likelihood Ratio Test.

Information criteria (AIC, BIC, and aBIC) consistently decreased as the number of profiles increased, indicating progressively better model fit. Specifically, the 2-profile model showed AIC = 20560.075, BIC = 20859.070, aBIC = 20627.400; the 3-profile model decreased to AIC = 19431.435, BIC = 19832.826, aBIC = 19521.817; the 4-profile model further dropped to AIC = 19077.146, BIC = 19580.932, aBIC = 19190.584; and the 5-profile model had the lowest values: AIC = 18921.803, BIC = 19527.985, aBIC = 19058.299.

Regarding model comparison tests, the Lo–Mendell–Rubin (LMR) adjusted likelihood ratio test was significant for both the 2-profile (*p* < 0.001) and 3-profile models (*p* = 0.006), indicating that adding profiles significantly improved model fit. However, the LMR test was non-significant for the 4-profile (*p* = 0.182) and 5-profile models (*p* = 0.789), suggesting no meaningful improvement beyond three profiles. The Bootstrap Likelihood Ratio Test (BLRT) was significant for all models (*p* < 0.001), but this test tends to favor models with more profiles in larger samples, so results were interpreted alongside other criteria.

Classification quality was high across all models, with entropy values ranging from 0.923 to 0.952, indicating good classification accuracy. The 2-profile model had an entropy of 0.949, the highest entropy was found in the 3-profile model (0.952), while the 4-profile and 5-profile models showed slightly lower entropy (0.928 and 0.923, respectively). Regarding profile sizes, the 3-profile model had proportions of 41.0, 51.2, and 7.8%, all exceeding the 5% minimum threshold for meaningful interpretation. In contrast, the 5-profile model included a very small profile comprising only 2.0% of the sample, which is impractical.

Therefore, the 3-profile model was selected as the optimal solution. It significantly improved fit over the 2-profile model per the LMR test, had the highest entropy, and demonstrated reasonable profile distributions, balancing statistical fit and theoretical interpretability.

**Figure 2:**
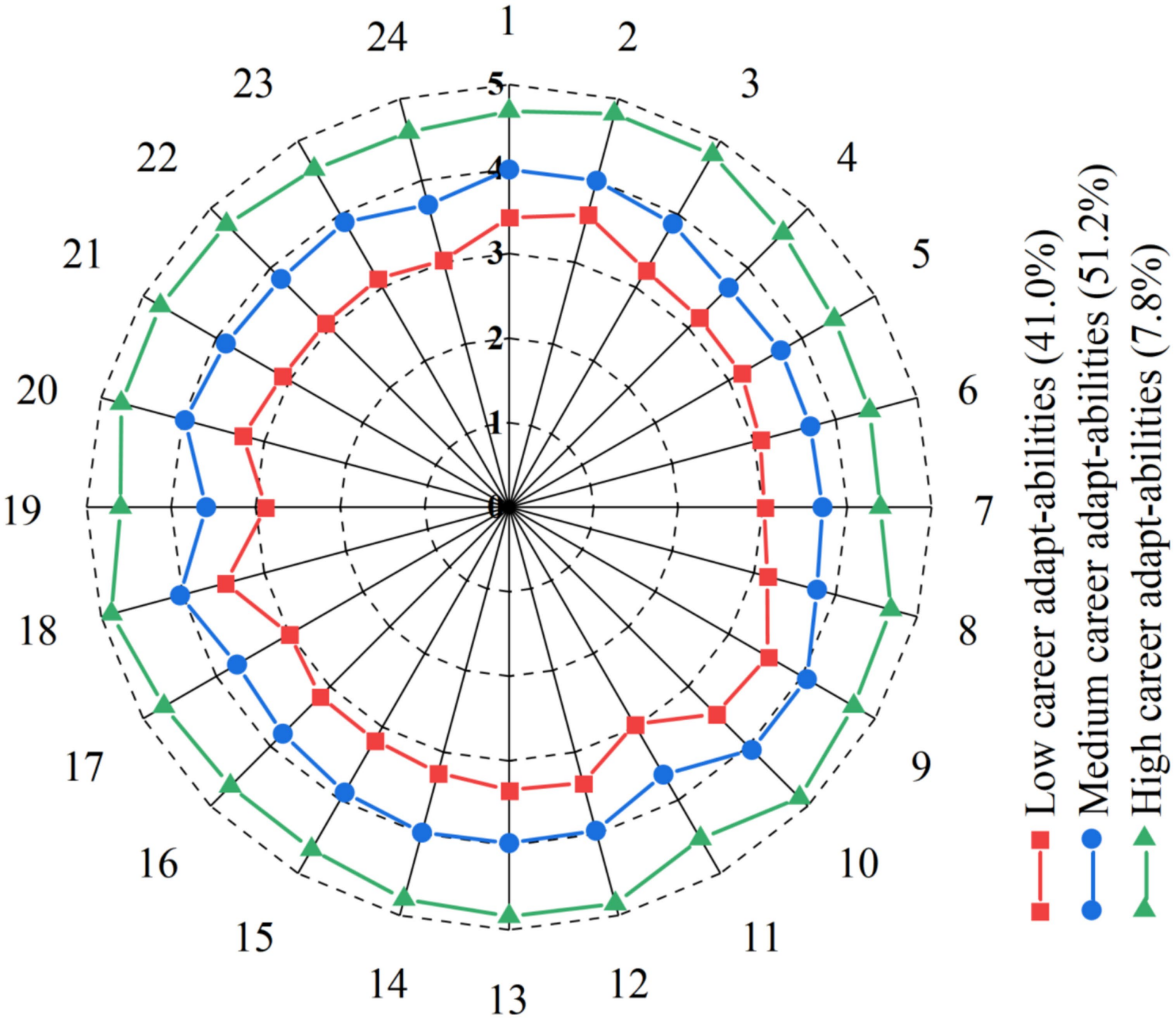
Profile of three potential subgroups of occupational resilience.

Based on the 3-profile model, three qualitatively distinct subgroups of career adapt-abilities were identified, as shown in Figure 2. By examining the conditional mean patterns across the 24 career adapt-abilities items and considering psychological characteristics, the profiles were labeled as “Low Career Adapt-Abilities Group,” “Moderate Career Adapt-Abilities Group,” and “High Career Adapt-Abilities Group.”

The Low Career Adapt-Abilities Group included 182 students (41.0% of the sample). This group showed relatively low levels across all four dimensions of career adapt-abilities: weak career concern, low career control, limited career curiosity, and poor career confidence. These students likely lack clear career planning and proactive preparation and tend to respond passively or avoidantly when facing career challenges and uncertainty.

The Moderate Career Adapt-Abilities Group was the largest, with 227 students (51.2%). They demonstrated moderate, balanced levels across all career adapt-abilities dimensions. These students possess basic career coping skills and some career awareness and reflection but have not yet developed a highly proactive career orientation.

The High Career Adapt-Abilities Group was the smallest, with 35 students (7.8%). This group exhibited significantly higher scores than the other two groups across all dimensions, showing strong career concern, high career control, robust career curiosity, and solid career confidence. These students actively plan their career development and demonstrate high flexibility and adaptability when facing career transitions and challenges.

The distribution of these profiles reveals substantial heterogeneity in career adapt-abilities development among English majors. Notably, over 40% fall into the low adapt-ability group, while fewer than 10% are in the high adapt-ability group. This pattern highlights the need for university English programs to place greater emphasis on cultivating and enhancing students’ career adapt-abilities.

### 4.7 Differences in AI anxiety and core self-evaluation across latent subgroups

#### 4.7.1 Hypothesis reminder (H4)

We examined whether the identified career adapt-abilities profiles differed systematically in core self-evaluations and AI anxiety, expecting a graded pattern whereby higher adapt-abilities profiles would show higher core self-evaluations and lower AI anxiety (H4).

#### 4.7.2 Mini-summary

Findings supported H4: profile membership was strongly associated with core self-evaluations and moderately associated with AI anxiety. The low adapt-abilities group exhibited the lowest self-evaluations and the highest AI anxiety, whereas the high adapt-abilities group showed the most favorable psychological pattern.

**Figure 3:**
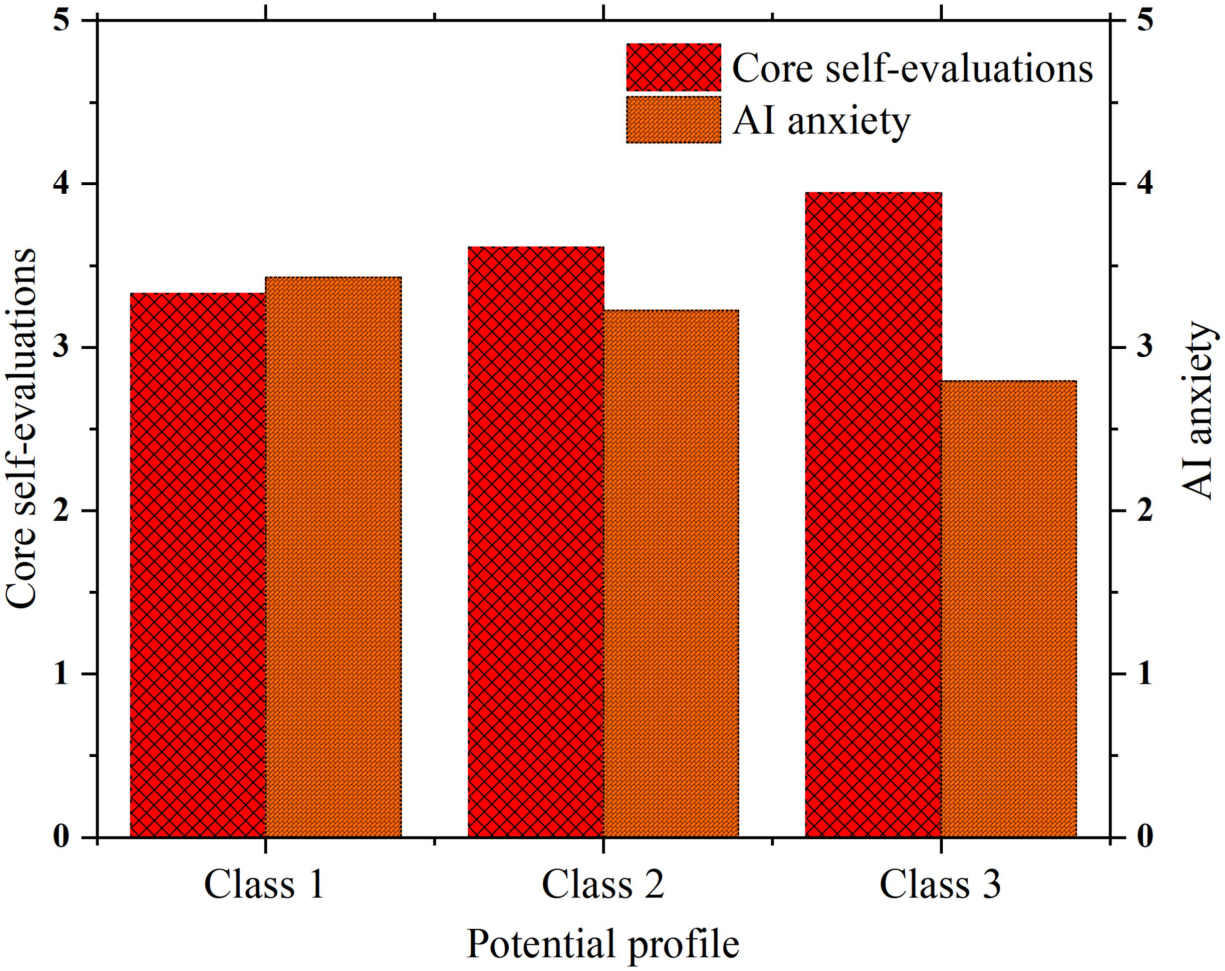
Analysis of differences in core self-rating and AI anxiety among latent subgroups. Class 1 = Low career adapt-abilities; Class 2 = Medium career adapt-abilities; Class 3 = High career adapt-abilities.

To examine whether English majors with different career adapt-abilities profiles significantly differ in core self-evaluation and AI anxiety, one-way ANOVAs were conducted using the latent profile classification as the independent variable, and core self-evaluation and AI anxiety as dependent variables. Results are shown in Table 8 and Figure 3.

**Table 8:** One-way ANOVA results for core self-evaluation and AI anxiety across latent subgroups.

Latent subgroups	Core self-evaluation		AI anxiety	
	*M*	SD	*M*	SD
Low career adapt-abilities	3.327	0.306	3.429	0.578
Medium career adapt-abilities	3.613	0.351	3.226	0.685
High career adapt-abilities	3.945	0.619	2.792	0.921
*p*	<0.001		<0.001	
*F*	57.593		14.695	
*η*^2^	0.207		0.062	

For core self-evaluation, there were highly significant differences among the three profile groups (*F* = 57.593, *p* < 0.001, *η*^2^ = 0.207). The effect size *η*^2^ = 0.207 indicates that profile membership explains about 20.7% of the variance in core self-evaluation, representing a large effect. Specifically, the Low Career Adapt-Abilities Group had the lowest core self-evaluation (*M* = 3.327, SD = 0.306), the Moderate group was intermediate (*M* = 3.613, SD = 0.351), and the High group had the highest scores (*M* = 3.945, SD = 0.619). Bonferroni *post hoc* tests showed all pairwise differences were statistically significant (*p* < 0.001), indicating a clear stepwise increase in core self-evaluation as career adapt-abilities level rises. This finding further confirms the positive predictive effect of career adapt-abilities on core self-evaluation found in the variable-centered analysis and reveals how this relationship manifests across distinct subgroups.

Regarding AI anxiety, significant differences were also found among the profiles (*F* = 14.695, *p* < 0.001, η^2^ = 0.062). The effect size *η*^2^ = 0.062 suggests profile membership explains about 6.2% of the variance in AI anxiety, a moderate effect. The Low Career Adapt-Abilities Group showed the highest AI anxiety (*M* = 3.429, SD = 0.578), followed by the Moderate group (*M* = 3.226, SD = 0.685), with the High group exhibiting the lowest anxiety (*M* = 2.792, SD = 0.921). *Post hoc* tests revealed significant differences between the Low and High groups (*p* < 0.001), and between the Low and Moderate groups (*p* < 0.05). These results align with theoretical expectations, suggesting that students with higher career adapt-abilities are better equipped to cope with perceived career threats posed by AI technologies, maintaining lower anxiety levels.

Notably, the High Career Adapt-Abilities Group’s mean AI anxiety score of 2.792 falls below the scale midpoint of 3, indicating relatively low anxiety, while the Low group’s mean of 3.429 is clearly above the midpoint, highlighting a pronounced AI anxiety issue for that subgroup. Additionally, the High group showed a larger standard deviation in AI anxiety (SD = 0.921), reflecting greater individual variability within this group, possibly due to differences in specific coping strategies employed to handle AI-related challenges.

### 4.8 Summary of hypothesis testing

Table 9 provides an integrated summary of all hypothesis tests conducted in this study.

**Table 9:** Summary of hypothesis testing results.

Hypothesis	Description	Analysis method	Key statistics	Result
H1	Career adapt-abilities significantly and negatively predict AI anxiety	PROCESS Model 4	*β* = −0.245, *p* < 0.001; Total effect = −0.463	Supported
H2	Career adapt-abilities → Core self-evaluations → AI anxiety	Bootstrap mediation (5,000 samples)	Indirect effect = −0.111, 95% CI [−0.208, −0.007];	Supported
H3	Distinct latent subgroups of career adapt-abilities exist	Latent Profile Analysis	3-profile solution; LMR *p* = 0.006; Entropy = 0.952	Supported
H4	Latent subgroups differ in core self-evaluations and AI anxiety	One-way ANOVA	Core self-evaluations: *F* = 57.593, *p* < 0.001, *η*^2^ = 0.207; AI anxiety: *F* = 14.695, *p* < 0.001, *η*^2^ = 0.062	Supported

## 5 Discussion

### 5.1 Person-centered analysis

Through latent profile analysis, three stable career adapt-abilities patterns were identified, highlighting significant heterogeneity in the psychological resource structures of English majors. This result challenges the traditional variable-centered assumption of homogeneity, indicating that career adapt-abilities cannot be fully explained by scores on individual dimensions alone; rather, the structured configuration of its four dimensions better reflects the true psychological functioning. For example, the low-profile group showed consistently weak career concern, control, curiosity, and confidence, indicating an overall low-resource system; in contrast, the high-profile group exhibited highly coordinated and elevated resources across all four dimensions. These structural differences suggest that career adapt-abilities should be viewed as a dynamic system formed through the synergy of multiple resource dimensions, rather than a simple linear sum of individual scores.

Further analysis showed that the high career adapt-abilities group had the highest core self-evaluation, followed by the moderate group, with the low group scoring lowest. This aligns with previous variable-centered research demonstrating a positive association between career adapt-abilities and core self-evaluation (Xu and Yu, 2021). A possible explanation comes from Social Cognitive Career Theory, which posits that successful experiences and positive feedback during career development are internalized as stable self-beliefs through enhanced self-efficacy (Lent, 2020; Fu et al., 2023). Individuals with high career adapt-abilities are more likely to gain success experiences when facing career challenges, which solidify the core belief “I am capable and valuable,” a central component of core self-evaluation. Thus, students with higher career adapt-abilities tend to have more positive self-concepts.

Regarding AI anxiety, the low career adapt-abilities group showed the highest anxiety levels, while the high group had the lowest. This supports the theoretical hypothesis of a negative relationship between career adapt-abilities and technology anxiety (Zhang et al., 2019; Fang and Xu, 2025). On one hand, career adapt-abilities serves as a vital psychological resource for coping with career uncertainty and buffers the negative effects of external threats on mental health (Gupta and Shagirbasha, 2025). On the other hand, individuals lacking career adapt-abilities resources may feel powerless when facing technological displacement threats, leading to negative appraisals that trigger and sustain anxiety (Akturan et al., 2025). Therefore, career adapt-abilities and AI anxiety may interact, with low career adapt-abilities students caught in a “resource depletion–anxiety escalation” negative spiral.

Moreover, the low career adapt-abilities group was characterized by significant deficits in career concern and confidence and exhibited notably higher AI anxiety than the moderate and high groups. This suggests that lack of career foresight and coping confidence may play key roles in exacerbating technology anxiety (Skrbiš and Laughland-Booÿ, 2019). Research on substance addiction shows that psychological resource deficits provoke negative emotional responses due to imbalances between threat perception and resource perception during cognitive appraisal (Palmwood and McBride, 2019). Our findings imply similar psychological mechanisms may underlie the relationship between career adapt-abilities and technology anxiety: when individuals lack necessary career psychological resources to face AI-related challenges, anxiety and worry intensify significantly.

### 5.2 Variable-centered analysis

This study confirmed a significant negative predictive effect of career adapt-abilities on AI anxiety, consistent with prior research on the protective effects of career psychological resources (Duan et al., 2025). Our study validates this proposition in the context of rapidly evolving AI technologies and further emphasizes the crucial role of this resource in maintaining students’ psychological stability amid technological displacement risks. Career adapt-abilities reduces cognitive threat perception of AI by enhancing coping strategies and future planning awareness, thereby effectively lowering anxiety (Johnston, 2018).

More importantly, this study revealed that core self-evaluation partially mediates the relationship between career adapt-abilities and AI anxiety, deepening the integrated explanatory framework of Conservation of Resources Theory and Career Construction Theory. Core self-evaluation effectively transforms accumulated career resources into stronger self-efficacy and self-worth affirmation (Chang et al., 2012). Students with higher career adapt-abilities are more likely to gain positive experiences during career tasks, which over time internalize into higher core self-evaluation. Improved core self-evaluation then reduces intrusive threat information and enhances control over future uncertainty, alleviating AI anxiety (Shen et al., 2024). This chain mechanism theoretically offers a key insight into the psychological processes underlying technology anxiety.

However, career adapt-abilities still showed a significant direct effect on AI anxiety, indicating core self-evaluation does not fully explain their relationship. One possible explanation is that career adapt-abilities dimensions related to agency and future orientation (e.g., concern and control) directly buffer threat perceptions (Jia et al., 2022). Students with high career adapt-abilities tend to proactively plan responses to AI impacts, actively acquire new knowledge, and maintain career control, directly reducing perceived technological threats. Thus, career adapt-abilities may operate via both cognitive and behavioral pathways, suggesting future research explore multi-level mechanisms.

### 5.3 Critical reflection on effect sizes and hypothesis refinement

While all four hypotheses received statistical support, a candid assessment of the effect sizes reveals important limitations that warrant careful consideration and suggest directions for theoretical refinement.

#### 5.3.1 Acknowledging modest effect sizes

The mediation model explained only 14.1% of the variance in AI anxiety, and the indirect effect through core self-evaluations accounted for 24% of the total effect. In the person-centered analysis, profile membership explained 6.2% of AI anxiety variance, representing a moderate rather than large effect. These findings, while statistically significant, indicate that career adapt-abilities and core self-evaluations constitute only part of the psychological equation underlying AI anxiety among English majors.

Several considerations help contextualize these effect sizes. First, AI anxiety is a complex psychological response influenced by multiple factors at individual, institutional, and societal levels (Frenkenberg and Hochman, 2025). Personality traits such as trait anxiety and neuroticism, institutional factors including curriculum design and career guidance quality, and macro-level factors such as labor market conditions and media representations of AI all likely contribute to AI anxiety (Kaya et al., 2024). The modest effect sizes observed in this study reflect the reality that career adapt-abilities, while important, operate alongside numerous other determinants. Second, in applied psychology research, effect sizes in the range observed here are not uncommon when examining single predictor-outcome relationships within complex phenomena. Cohen’s guidelines suggest that η^2^ values between 0.06 and 0.14 represent medium effects, and our findings fall within or near this range. Third, from a practical standpoint, even modest effect sizes can translate into meaningful differences at the population level.

#### 5.3.2 Revisiting theoretical assumptions

The modest effect sizes invite reconsideration of some theoretical assumptions underlying this study.

Career adapt-abilities as a primary protective resource. The original conceptualization positioned career adapt-abilities as a key psychological resource buffering AI anxiety. While the data support this relationship, the limited explanatory power suggests that career adapt-abilities may be better conceptualized as one component within a broader constellation of protective resources. Future theoretical models should adopt a more integrative approach, examining how career adapt-abilities interact with other resources such as social support, institutional support, AI self-efficacy, and growth mindset to collectively influence AI anxiety.

Core self-evaluations as the primary mediating mechanism. The partial mediation finding (24% indirect effect) indicates that core self-evaluations represent only one of potentially multiple pathways through which career adapt-abilities influence AI anxiety. The substantial direct effect (76% of total effect) suggests the existence of other mediating mechanisms not captured in the current model. Possible alternative mediators include career decision-making self-efficacy, future time perspective, perceived employability, proactive coping strategies, and AI-specific self-efficacy. Future research should test multiple parallel mediators to develop a more comprehensive understanding of the mechanisms linking career adapt-abilities to AI anxiety.

Homogeneity of effects across contexts. The current study assumed that the relationships among variables operate similarly across different contexts. However, the effect of career adapt-abilities on AI anxiety may be moderated by contextual factors such as perceived proximity to labor market entry, exposure to AI in coursework, quality of institutional career services, and regional labor market conditions. The relatively modest effects observed may partly reflect heterogeneity in these contextual factors that was not modeled.

#### 5.3.3 Reinterpreting the high career adapt-abilities group findings

An intriguing finding warranting further discussion is the relatively large standard deviation in AI anxiety within the high career adapt-abilities group (SD = 0.921, compared to 0.578 in the low group). This heterogeneity suggests that high career adapt-abilities do not uniformly protect against AI anxiety. Some highly adaptable students may experience elevated anxiety precisely because their strong career concern leads them to attend more closely to AI-related occupational threats. This pattern aligns with the “awareness-anxiety paradox” observed in other domains: greater awareness and engagement with a potential threat can sometimes increase rather than decrease anxiety, at least in the short term. Future research should examine whether specific configurations of career adapt-abilities dimensions (e.g., high concern combined with lower confidence) produce differential anxiety outcomes, and whether the relationship between career adapt-abilities and AI anxiety is curvilinear rather than strictly linear.

#### 5.3.4 The limited role of AI usage habits

The non-significant relationship between AI usage habits and AI anxiety represents a finding that diverges from intuitive expectations. One might expect that greater familiarity with AI tools would reduce anxiety through exposure and mastery experiences. The absence of this relationship suggests several possibilities. First, mere exposure to AI tools may be insufficient to reduce anxiety about AI’s occupational implications; the anxiety may be driven more by abstract concerns about replaceability than by direct experiences with AI tools. Second, the categorization of AI usage habits in this study may have been too crude to capture meaningful variations in how students engage with AI. Future research should distinguish between passive use of AI tools, active exploration and skill development, and critical engagement with AI capabilities and limitations. Third, this finding may indicate that AI anxiety among English majors is primarily anticipatory and occupationally oriented rather than stemming from direct negative experiences with AI tools.

### 5.4 Theoretical implications and dialog with existing literature

This study makes several distinctive contributions to the understanding of technology-related anxiety in the context of AI-driven labor market transformation, advancing both theoretical frameworks and empirical knowledge in vocational psychology. Bridging Career Construction Theory and Conservation of Resources Theory in the AI Context. A primary theoretical contribution lies in the integration of Career Construction Theory and Conservation of Resources Theory to explain the formation mechanisms of AI anxiety among English majors. While previous research has applied these theories separately to career development outcomes (Wang et al., 2021; Rudolph et al., 2019), this study demonstrates their complementary explanatory power in the specific context of technological displacement threats. Career Construction Theory emphasizes career adapt-abilities as readiness for vocational transitions, yet it does not fully articulate the cognitive mechanisms through which these resources translate into psychological well-being. By incorporating Conservation of Resources Theory, this study reveals that core self-evaluations serve as the critical cognitive transformation mechanism, converting accumulated career resources into stable self-beliefs that buffer against external threats. This integrated framework extends beyond the general propositions of both theories, offering a more nuanced understanding of how psychological resources operate under conditions of technological uncertainty.

Contextualizing Findings within the Broader Literature on Technological Unemployment Anxiety. The findings contribute meaningfully to the emerging literature on psychological responses to AI-induced occupational disruption. Prior studies have predominantly focused on technology workers and general adult populations, documenting significant associations between AI exposure and job insecurity, career uncertainty, and psychological distress. For instance, research by Brougham and Haar (2018) found that awareness of smart technology significantly predicted job insecurity across various industries, while Kaya et al. (2024) demonstrated that perceived AI threat was associated with increased anxiety among knowledge workers. The present study extends this literature by examining a previously understudied population—humanities students whose professional identity is directly challenged by AI language technologies. Our finding that career adapt-abilities negatively predict AI anxiety resonates with broader research showing that psychological resources buffer technology-related stress, yet the magnitude of this relationship appears somewhat lower than that observed in technology-intensive occupations. This difference may reflect the unique position of English majors, who face not merely skill obsolescence but a fundamental challenge to the epistemological value of their expertise in an era when AI systems can generate, translate, and analyze language at unprecedented scales.

Situating Findings in the Discourse on AI and the Future of Language Professions. The study’s findings also speak to broader debates about the future of language-related professions in the AI era. The World Economic Forum has classified translation, content creation, and language education as high-risk occupations for automation (Abdullah, 2025), yet the human dimensions of these roles—cultural interpretation, ethical judgment, emotional intelligence, and contextual sensitivity—remain difficult to automate. Our finding that students who perceive their future careers as related to AI technologies show marginally higher anxiety (though not statistically significant at *p* = 0.075) suggests that awareness of technological relevance may operate as a double-edged sword, heightening concern even as it potentially motivates adaptive behavior. This nuanced pattern echoes arguments by scholars such as Frey and Osborne (2017), who have noted that subjective perceptions of automation risk often diverge from objective assessments, with psychological responses shaped more by perceived than actual substitutability. For English majors specifically, the challenge lies not only in acquiring new technical competencies but in reconstructing professional identities that position human linguistic expertise as complementary to, rather than competitive with, AI capabilities.

Our research results surpass those of previous studies, as shown in Table 10.

**Table 10:** Summary of theoretical contributions of this study.

Contribution	How it surpasses prior studies
Population focus	First study to examine career adapt-abilities and AI anxiety specifically among humanities students whose core competencies are challenged by AI.
Theoretical integration	Novel integration of Career Construction Theory and Conservation of Resources Theory in AI context.
Mediating mechanism	First empirical test of core self-evaluations as mediator between career resources and technology-specific anxiety.
Person-centered approach	First latent profile analysis linking career adapt-abilities profiles to AI anxiety outcomes.
Context specificity	Extends Western-developed frameworks (Judge et al., 2003; Hobfoll, 1989; Savickas, 2013) to Chinese context and AI-specific stressor.
Developmental timing	Examines pre-career individuals during professional identity formation, unlike prior studies of employed adults.

### 5.5 Practical implications

This study’s findings have important practical guidance for reforming English major education and psychological services in universities. First, the three latent career adapt-abilities subgroups provide empirical support for stratified and targeted educational interventions. For the over 40% low career adapt-abilities group, educators should prioritize cultivating career adapt-abilities through career planning courses, counseling workshops, and alumni career talks to systematically enhance career concern, control, curiosity, and confidence. For the moderate group, interventions should consolidate existing resources and promote progression to higher levels, while for the high group, efforts should focus on translating psychological strengths into concrete human-AI collaboration and technical integration skills. Such differentiated interventions based on profile types can improve precision in resource allocation and intervention effectiveness.

The mediating role of core self-evaluation suggests that cultivating career adapt-abilities should be accompanied by fostering and optimizing students’ self-concept. Educators can create opportunities for skill demonstration, provide positive feedback, and promote accumulation of success experiences to help students build positive recognition of their professional value and abilities. Especially in the context of rapid AI development, students need guidance to recognize the irreplaceability of human language professionals in cultural understanding, situational judgment, creative expression, and ethical decision-making, thereby cognitively reconstructing their professional identity. Psychological counseling should also address core self-evaluation status, using evidence-based interventions like cognitive-behavioral therapy for students showing low self-efficacy, high neuroticism, or external locus of control to help them adjust maladaptive self-cognitions.

The findings also inform the design of AI literacy education in universities. The lack of a significant link between AI usage habits and AI anxiety suggests that merely increasing students’ exposure to AI tools does not automatically reduce anxiety. Effective AI literacy education should go beyond technical skills to incorporate psychological adaptation dimensions. For example, alongside teaching AI tool use, courses can discuss AI’s limitations and boundaries to foster rational human-machine relationship understanding; through case studies and simulations, they can demonstrate practical collaboration scenarios between language professionals and AI, transforming abstract technical threats into concrete opportunities for skill development. This integrated psychological adjustment approach can more effectively help students shift from “technology anxiety” to “technology empowerment.”

At the macro level, the study indicates that English major curricula and training goals need adaptive adjustments. Traditional English education focuses on language skills, but under the reshaping influence of AI on language services, this single-focus model faces challenges. Our results highlight career adapt-abilities as a cultivable psychological resource critical for coping with technological changes and maintaining mental health. Therefore, universities should systematically incorporate career adapt-abilities development into English major programs through curriculum reform, practical projects, and career guidance to foster psychological resilience and adaptability amid career uncertainty. Meanwhile, university mental health services should recognize AI anxiety as an emerging issue and develop targeted assessments and interventions to support students struggling with technology-related anxiety.

The training prospects for English graduates should be highlighted more explicitly given the rapid diffusion of generative AI in language-related tasks. Our findings suggest that interventions should not only focus on technical upskilling, but also on building psychosocial resources that help students navigate uncertainty and occupational change. Programs can embed AI into core skill modules (translation, writing, interpreting, business communication) by teaching students to (a) formulate effective prompts, (b) evaluate output quality, bias, and factuality, and (c) revise and post-edit AI-generated drafts to meet professional standards. This reframes AI from a replacement threat into a productivity tool and clarifies the unique value of human linguistic judgment. Since career adapt-abilities are linked to lower AI anxiety, departments can implement structured activities aligned with the four adapt-ability dimensions (concern, control, curiosity, and confidence), such as career scenario planning, goal-setting workshops, informational interviews with AI-affected occupations, and mastery-based project scaffolding. These activities can be integrated into first-year seminars and capstone projects rather than offered only as optional counseling. To translate training into labor-market advantage, programs can require AI-aware portfolios (e.g., bilingual content strategy, localization projects, terminology management, and AI-assisted editing logs) and strengthen partnerships with employers for internships in roles where English graduates can leverage human strengths (cross-cultural communication, content governance, quality assurance, and client-facing mediation).

### 5.6 Limitations and future research directions

While this study advances understanding of AI anxiety formation mechanisms among English majors, several limitations should be acknowledged, each pointing toward productive directions for future research and offering reflections on the broader implications of our findings.

The cross-sectional design precludes causal inferences about the temporal ordering of career adapt-abilities, core self-evaluations, and AI anxiety. Although the theoretical model posits that career adapt-abilities precede and shape core self-evaluations, which in turn influence AI anxiety, alternative causal sequences are plausible. Students experiencing high AI anxiety may, for instance, become less engaged in career planning activities, leading to diminished career adapt-abilities over time. Longitudinal studies employing latent growth curve modeling or cross-lagged panel designs would enable examination of these dynamic relationships, tracking how career adapt-abilities profiles evolve and whether profile transitions predict changes in AI anxiety. Such designs would also permit investigation of critical developmental windows during which interventions might be most effective.

The reliance on self-report measures introduces potential biases, including social desirability effects and shared method variance. While Harman’s single-factor test suggested that common method bias was not severe, more rigorous approaches—such as marker variable techniques or multi-trait multi-method designs—would strengthen confidence in the findings. Future studies might incorporate objective indicators of career adapt-abilities (e.g., documented career planning activities, internship participation) and physiological measures of anxiety (e.g., cortisol levels, heart rate variability) to triangulate self-report data.

The sample, drawn from four universities in Sichuan Province, provides reasonable regional diversity but may not fully represent English majors across China’s varied educational and economic contexts. Universities in eastern coastal regions, where the language services industry is more developed and AI adoption more visible, may produce students with different career adapt-abilities profiles and anxiety patterns. Cross-regional comparisons would clarify how local labor market conditions moderate the relationships observed in this study. Furthermore, international comparative research could examine whether the protective effect of career adapt-abilities operates similarly across cultural contexts with different educational philosophies, employment structures, and attitudes toward AI technologies.

Unexplored Antecedents and Contextual Factors. This study focused on the consequences of career adapt-abilities profiles but did not examine their antecedents. Understanding why some students develop high career adapt-abilities while others remain in low-resource configurations is essential for designing effective interventions. Potential antecedent factors include personality traits (particularly openness to experience and conscientiousness), family socioeconomic background, quality of career guidance received, and exposure to successful role models in evolving language professions. Qualitative research exploring students’ subjective experiences of AI-related occupational uncertainty would complement the quantitative findings, illuminating the meaning-making processes through which students interpret technological change and construct career narratives.

A critical next step is developing and rigorously evaluating interventions designed to enhance career adapt-abilities and reduce AI anxiety among English majors. The person-centered findings suggest that interventions should be differentiated according to students’ baseline career adapt-abilities profiles. For students in the low-resource group, foundational interventions building career concern and confidence may be most urgent, whereas students in the moderate group may benefit from consolidation and enhancement strategies. Randomized controlled trials comparing different intervention approaches—such as career counseling workshops, AI literacy programs integrated with psychological support, and mentoring from professionals who have successfully navigated technological transitions—would provide evidence to guide educational practice.

Finally, the study’s findings invite broader reflection on the relationship between AI technologies and human expertise in language-related domains. The moderate level of AI anxiety observed among English majors suggests neither overwhelming panic nor complacent dismissal of technological change. This measured response may indicate that students recognize both the challenges and opportunities presented by AI, even if they lack confidence in their ability to navigate these changes successfully. Educational institutions bear responsibility for helping students develop not only technical competencies for human-AI collaboration but also the psychological resilience to adapt to ongoing technological evolution. The career adapt-abilities framework provides a valuable lens for conceptualizing this resilience, but institutions must also address structural factors—such as curriculum design, industry partnerships, and career services—that shape the contexts within which students develop their career resources. In this sense, the study’s findings point beyond individual-level interventions toward systemic transformations in how humanities education prepares students for an AI-infused professional landscape.

## 6 Conclusion

This study, adopting both variable- and person-centered perspectives, revealed the key role of career adapt-abilities in alleviating AI anxiety among English majors. Variable-centered analysis showed that career adapt-abilities not only directly reduces AI anxiety but also indirectly protects through enhancing core self-evaluation. Person-centered analysis identified three career adapt-abilities profiles with systematic differences in core self-evaluation and AI anxiety; the low adapt-ability group faced the highest risk, while the high group had the strongest psychological advantages. Overall, the findings confirm career adapt-abilities as a crucial psychological resource for coping with AI-driven occupational pressures and demonstrate that its effect mechanism partly relies on core self-evaluation. The marked internal heterogeneity of career adapt-abilities highlights the necessity of stratified and precise interventions in career education and psychological support. This study offers new empirical evidence for understanding the formation mechanisms and protective factors of technology anxiety among college students and provides practical insights for transforming and adjusting talent development in universities in the AI era.

## Data Availability

The raw data supporting the conclusions of this article will be made available by the authors, without undue reservation.

## Footnotes

1
https://www.credamo.com/
